# Supplementary material for: Association of sleep abnormalities in older adults with risk of developing Parkinson’s disease
Source: Sleep. 2022 Aug 29;45(11):zsac206. doi: 10.1093/sleep/zsac206 (PMC9644115; doi:10.1093/sleep/zsac206)
Supplement: zsac206_suppl_Supplementary_Material [file zsac206_suppl_supplementary_material.docx]

Title: Association of sleep abnormalities in older adults with risk of developing Parkinson’s disease

Author: Abidemi I. Otaiku^1,2^ BMBS BSc AKC

1 Department of Neurology, Birmingham City Hospital, Birmingham, UK

2 Centre for Human Brain Health, University of Birmingham, Birmingham, UK

Correspondence:

Dr Abidemi Otaiku, Department of Neurology, Birmingham City Hospital, Dudley Road,

Birmingham B18 7QH, UK. 0121 554 3801. a.otaiku@nhs.net

Table S1. Odds ratios and 95% CI for incident PD according to self-reported sleep apnoea diagnosis

| **Physician-diagnosed sleep apnoea** |  |  |  |
| --- | --- | --- | --- |
|  | Yes | No | Don’t know |
| PD cases [n (%)] | 5 (3.4) | 63 (2.5) | 2 (3.0) |
| N | 149 | 2555 | 66 |
| Age + clinic adjusted | 1.28 (0.5, 3.2) | 1 [reference] | 1.14 (0.3, 4.8) |
| Multivariable adjusted^a^ | 1.04 (0.4, 2.7) | 1 [reference] | 1.00 (0.2, 4.4) |

^a^ Adjusted for age, clinic site, race, education, smoking status, diabetes, hypertension, depressive symptoms, cognitive function, daytime sleepiness, physical activity levels, body mass index, caffeine intake, and psychotropic medication use.

**P* < 0.05

***P* < 0.01

****P* < 0.001
